# Kinetic and Structural Characterization of the Effects of Membrane on the Complex of Cytochrome *b*_5_ and Cytochrome *c*

**DOI:** 10.1038/s41598-017-08130-7

**Published:** 2017-08-10

**Authors:** Katherine A. Gentry, Elke Prade, Carlo Barnaba, Meng Zhang, Mukesh Mahajan, Sang-Choul Im, G. M. Anantharamaiah, Satoshi Nagao, Lucy Waskell, Ayyalusamy Ramamoorthy

**Affiliations:** 1 Biophysics Program, University of Michigan, Ann Arbor, MI 48109 USA; 2 Department of Chemistry, University of Michigan, Ann Arbor, MI 48109 USA; 3 Department of Anesthesiology, University of Michigan, and Veterans Affairs Medical Center, Ann Arbor, Michigan 48105 USA; 4 Department of Medicine, UAB Medical Center, Birmingham, Alabama 35294 USA; 5 Graduate School of Material Science, Nara Institute of Science and Technology, 8916-5 Takayama, Ikoma, Nara 630-0192 Japan

## Abstract

Cytochrome *b*_5_ (cyt*b*_5_) is a membrane protein vital for the regulation of cytochrome P450 (cytP450) metabolism and is capable of electron transfer to many redox partners. Here, using cyt *c* as a surrogate for cytP450, we report the effect of membrane on the interaction between full-length cyt*b*_5_ and cyt c for the first time. As shown through stopped-flow kinetic experiments, electron transfer capable cyt*b*_5_ - cyt c complexes were formed in the presence of bicelles and nanodiscs. Experimentally measured NMR parameters were used to map the cyt*b*_5_-cyt c binding interface. Our experimental results identify differences in the binding epitope of cyt*b*_5_ in the presence and absence of membrane. Notably, in the presence of membrane, cyt*b*_5_ only engaged cyt c at its lower and upper clefts while the membrane-free cyt*b*_5_ also uses a distal region. Using restraints generated from both cyt*b*_5_ and cyt c, a complex structure was generated and a potential electron transfer pathway was identified. These results demonstrate the importance of studying protein-protein complex formation in membrane mimetic systems. Our results also demonstrate the successful preparation of novel peptide-based lipid nanodiscs, which are detergent-free and possesses size flexibility, and their use for NMR structural studies of membrane proteins.

## Introduction

Microsomal cytochrome *b*_5_ (cyt*b*_5_) is a membrane-bound protein that is involved in electron transport to several redox partners including cytochrome P450 (cytP450), several oxygenases and desaturases, and cytochrome *b*_5_ reductase^1, 2^. Cytochrome *b*_5_ interactions ensure function/activation of cytP450, which plays a vital role in cellular metabolism, including the metabolism of over 70% of drugs in the current market and has been implicated in heart diseases and breast and prostate cancers^3–5^. Because of its membrane-bound native state and the transient nature of cyt*b*_5_-cytP450 complex formation, there is relatively little structural insight into this disease relevant complex, especially when compared to the interactions of soluble proteins involved in electron transport.

For decades, it has puzzled investigators how cyt*b*_5_ is able to enhance, reduce, or exert no effect on cytP450 metabolism depending on the substrate and the isoform of cytP450 involved^6–10^. Recent studies with microsomal cytP450 have helped elucidate how cyt*b*_5_ causes it apparent contradictory effects^6, 11, 12^, but few studies have been undertaken in the presence of membrane. There are numerous challenges to obtaining high-resolution structural insights into membrane-bound cytP450 due to its size and the tendency for the full-length protein to aggregate in solution, which make it difficult to use the traditional solution NMR experiments^10, 13, 14^. Because of these reasons, most reported studies in the literature have focused only on the soluble domain of cytP450, lacking the N-terminal transmembrane domain. On the other hand, previous studies have used cytochrome *c* (cyt *c*) as a model for cytP450 in probing the structural interactions between cyt *c* with other proteins like cyt*b*_5_ or CPR^15–19^. Cyt *c* has several similarities to cytP450 including: a similar, overlapping binding domain on cyt*b*_5_, an overall net positive charge integral for the initial protein-protein complex formation, and membrane has been suggested to promote or enhance activity of both proteins^20, 21^. Although cyt *c* is a much smaller protein (104 amino acids compared to 491 amino acids in cytP450 2B4), alignment of these two proteins reveal homologous amino acid sequences including known areas of cyt*b*_5_ interaction (Figure S1). Besides being a valid substitute, cyt *c* also has the advantage of being a well-behaved, NMR-friendly soluble protein. Structural and kinetic details of the electron-transfer complex of cytochrome *b*_5_ – cytochrome *c* (cyt *c*) have been the focus of many studies, including NMR spectroscopy^22^, MD simulations^23^, and mutagenesis studies^16, 18, 24^. While cyt *c* is not a major physiological electron transfer partner of cyt*b*_5_, it has been shown to be a productive electron transfer complex^25, 26^. Previous NMR studies have demonstrated that this complex exists in a 1:1 molar ratio in solution^27^. In this study, cyt *c* is used as a model for cyt P450 to examine the role that membrane plays in the formation of an electron transfer cyt*b*_5_-cyt*c* complex.

Most studies thus far have focused on the interaction of cyt *c* with the soluble domain of cyt*b*_5_ (without the C-terminal transmembrane (TM) domain) in the absence of membrane^15, 16, 28^. While several of these studies have shown that the truncated-cyt*b*_5_ is capable of slow electron transfer to bacterial soluble cytP450 s^29, 30^, others have shown truncated-cyt*b*_5_ to be incapable of transferring electrons to mammalian membrane-bound cytP450^30–32^. Full-length cyt*b*_5_ is necessary to fully understand this protein-protein interaction. Our recent studies have shown that lipid membrane plays an important role in the structural interactions between cyt*b*_5_ and cytP450^33–36^. Therefore, it is important to investigate the role of membrane on the interactions between cyt*b*_5_ and cyt *c*.

Studying membrane proteins is challenging due to their dynamic nature and difficulties encountered during expression and purification. Additionally, in the case of cyt*b*_5_, as with other single transmembrane helix proteins, the TM domain has been shown to increase the tendency to induce protein aggregation in solution. To combat these difficulties, we have used the full-length ~16-kDa rabbit cyt*b*_5_ in the presence of membrane mimetics which aid in the monomerization and stabilization of cyt*b*_5_ to probe how the inclusion of membrane affects the cyt*b*_5_-cyt c complex formation. Solution NMR experiments are used to probe the transient and dynamic protein-protein complex in a near-native membrane environment. NMR is well-suited for this study as it can provide residue specific details of cyt*b*_5_-cyt *c* in the timescale of the complex formation. Two different types of membrane mimetics are used in this study: isotropic bicelles and lipid nanodiscs. Isotropic bicelles have successfully been used to incorporate cyt*b*_5_ and perform NMR experiments to study its interaction with cytP450^33^. These isotropic bicelles have a planar lipid bilayer surrounded with detergents. Using bicelles as a membrane mimetic is preferable to the more traditional micelle as the micelle curvature could distort the structural folding of a membrane protein and the detergent could denature the embedded protein^37^. A benefit of using isotropic bicelles is that they tumble fast in the NMR time scale to enable the application of solution NMR experiments for structural studies^38^. The second membrane mimetic used in this study is lipid nanodiscs. Nanodiscs are composed of a lipid bilayer that is typically surrounded by a protein belt. Traditionally, this protein belt is the membrane scaffold protein (MSP)^39, 40^, but in this study we prepared nanodiscs using a short helical amphipathic peptide scaffold, referred to as 4 F^41^. This peptide has several advantages for our experiments compared to traditional MSP. Firstly, the nanodiscs can be prepared without the use of detergents, which are known to irreversibly inactivate proteins^37^. Secondly, a feature of peptide-based nanodisc is that the size is easily controlled through changing the lipid-to-peptide ratio. Lastly, the preparation allows for highly reproducible production of isotropic nanodiscs for solution NMR studies. In peptide-based nanodiscs, cyt*b*_5_ is readily monomerized and stable for weeks at room temperature^34^. As recent studies have identified, cyt *c* can undergo structural changes in the presence of cardiolipin^35, 36^, membrane mimetics used in these experiments were cardiolipin-free, such that cyt *c* would not interact with the membrane environment.

In this study, we investigated the interaction between full-length rabbit cyt*b*_5_ and full-length cyt *c* in different membrane mimetic environments, including lipid-free, isotropic bicelles, and lipid nanodiscs, utilizing solution NMR techniques. Our study provides further evidence that the interaction between these two redox partners is governed by the presence of lipid bilayers. While previous studies of truncated cyt*b*_5_ have identified productive binding, our results show an increased, dynamic interaction and changes in the residues most perturbed by complex formation as well as kinetic data demonstrating the formation of a productive complex.

## Results

### Incorporation of the cytb_5_-cyt c complex into membrane mimetics

**Figure 1:**
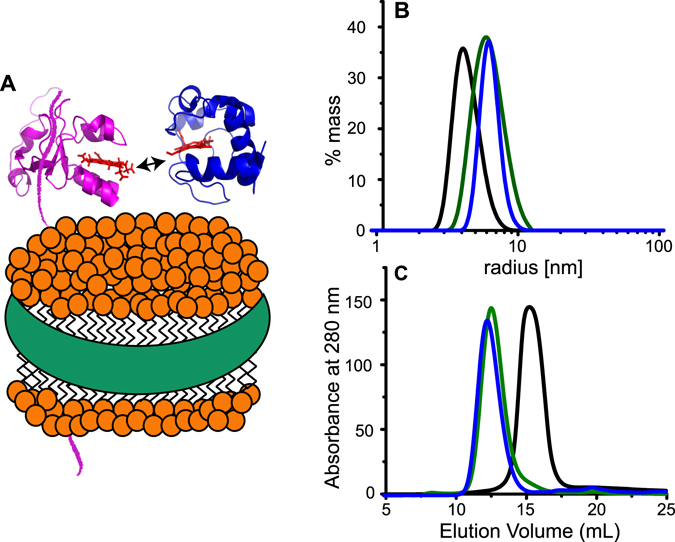
Reconstitution of cyt*b*_5_ – cyt c in lipid nanodiscs. (**A**) Schematic of the interaction between cyt*b*_5_ (magenta; PDB: 2M33) and cyt c (blue; PDB: 1HRC) in a lipid nanodisc. (**B**) Dynamic light scattering (DLS) was used to determine the size of the nanodiscs. Radii of empty 4F-DMPC-nanodiscs (black), cyt*b*_5_ in nanodiscs (green), and cyt*b*_5_ – cyt c in nanodiscs (blue). (**C**) Size exclusion chromatography (SEC) elution profiles of empty 4F-DMPC-nanodiscs (black) which eluted at 15.1 mL, cyt*b*_5_ in nanodiscs (green) which eluted at 12.6 mL, and cyt*b*_5_ – cyt c in nanodiscs (blue) which eluted at 12.3 mL.

In order to study the interaction between cyt*b*_5_ and cyt c, full-length cyt*b*_5_ was overexpressed, purified and characterized as reported previously^34, 42–44^. Three different cyt*b*_5_ samples were prepared in this study: cyt*b*_5_ in buffer (membrane-free cyt*b*_5_); cyt*b*_5_ in isotropic bicelles; and cyt*b*_5_ in lipid nanodiscs. Cyt*b*_5_ was added to DMPC/DHPC bicelles. While the isotropic bicelles stabilize the protein, they are not stable enough to be used in size exclusion chromatography. However, the monomerization and incorporation of cyt*b*_5_ in lipid nanodiscs can be monitored by Size Exclusion Chromatography (SEC) and Dynamic Light Scattering (DLS). Figure 1(B and C) (green trace) demonstrate that cyt*b*_5_ has been incorporated into the lipid nanodiscs in a homologous manner. After purification of the cyt*b*_5_ in lipid nanodiscs, cyt c was added to the sample and run through SEC and DLS experiments again (blue trace). These measurements show the complex formation between cyt*b*_5_ and cyt *c* in nanodiscs as indicated by the change in the size of the diameter fit to the DLS data and the elution profile from the SEC (Fig. 1). Cyt c does not interact with the nanodisc, but it only interacts cyt*b*_5_ (Figure S2).

### Formation of productive electron transfer complex between cytb_5_ and cyt c

**Figure 2:**
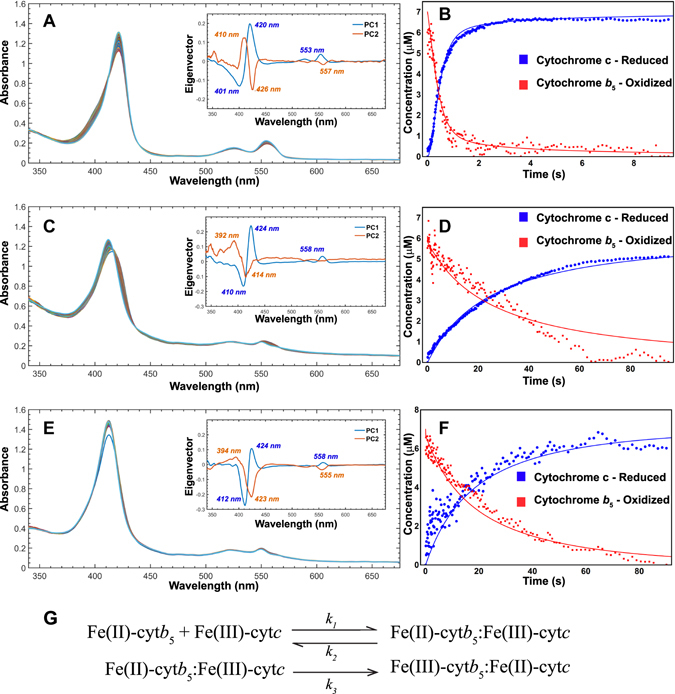
Spectral deconvolution and kinetic modeling reveal membrane environment dependent changes. Time-dependent absorption spectra of the redox couple are depicted for lipid-free (**A**), bicelles (**C**), and nanodiscs (**E**) mimetic membranes. The inset shows the eigenvectors associated with cyt *c* reduction (PC1) and cyt*b*_5_ oxidation (PC1); absorbance maxima are also indicated. The shift of λ_max_ as well as the peak intensity, an indicator of how far along the reaction has progressed, can be attributed to the presence of lipids. Numerical fittings are shown for lipid-free (**B**), bicelles (**D**), and nanodiscs **(F**). (**G**) Kinetic scheme of electron transfer between cyt*b*_5_ and cyt c. In the first reaction, oxidized cyt*b*_5_ and reduced cyt c form a complex with association rate *k*_1_ and dissociation rate *k*_2_. Once the first reaction has occurred then the second reaction occurs irreversibly with a rate of *k*_3_ of the electron transfer from oxidized cyt*b*_5_ to reduced cyt c giving oxidized cyt*b*_5_ and reduced cyt c.

Stopped flow kinetic experiments were performed to verify that the cyt*b*_5_-cyt *c* complex was active in all three preparations. The electron transfer rate was monitored between reduced cyt*b*_5_ and oxidized cyt *c*. Ferric cyt*b*_5_ was reduced by sodium dithionite and the reduction was monitored by UV-Vis spectrometry (Figure S3). Singular Value Decomposition (SVD) was applied to the difference spectra to deconvolute the principal spectral components (Fig. 2(A,C,E)). For the three samples, two eigenvectors were selected, which variance sum covered 82%, 69%, and 75% of total variance for lipid-free, bicelles, and nanodiscs, respectively. For bicelles, since the cumulative variance covered by the first two eigenvectors was slightly <70%, we used the scree plot to confirm that the first two components (PC1 and PC2) explained most of the variability in the data (Figure S4). The two components and their peak maxima and minima can be identified as either cyt*b*_5_ or cyt c based on the difference spectra for each preparation of cyt*b*_5_ (lipid-free, bicelles, and nanodiscs). Differences in the peak positions are attributed to the presence of lipids in the solution.

Kinetics traces corresponding to the oxidoreductive reactions (Fig. 2(B,D,F)) were modeled according to the scheme depicted in Fig. 2G. After formation of the complex between ferrous cyt*b*_5_ and ferric cyt *c* (*k*_1_ and *k*_2_), the electron transfer occurs (*k*_3_). We postulate that after the initial electron transfer no further reaction occurs, since it is not possible to distinguish between free and complexed cytochromes based on the absorption spectrum. Two observations were made: 1) the interaction between the redox partners is functional, and 2) the kinetic micro-rates indicate significant differences in the protein binding and electron transfer.

Table 1 reports the micro-rates determined from fitting the time-dependent kinetic traces with the numerical method. For lipid-free systems, the dissociation rate (*k*_2_) was fixed to zero, in order to allow the fitting to converge to a minimum. Bicelles and nanodiscs showed similar association and dissociation rates. Regarding electron transfer, the average micro-rate (*k*_3_) was 6.82 s^−1^ for lipid-free, 0.92 s^−1^ for bicelles, and 1.23 s^−1^ for nanodiscs cyt*b*_5_ samples. These results indicate that all three samples form functional complexes with membrane-free cyt*b*_5_ having the fastest electron transfer event while nanodiscs reconstituted cyt*b*_5_ has slightly faster rate than bicelles reconstituted cyt*b*_5_.

**Table 1:** Kinetic micro-rates obtained from numerical fitting of the stopped-flow time-time dependent traces using the kinetic scheme depicted.

	*k*_1_ (s^−1^)	*k*_2_ (s^−1^)	*k*_3_ (s^−1^)	*R* ^2^
**Lipid-free cytb5**	0.47 ± 0.01	~0^a^	6.82 ± 0.75	0.983
**Bicelle-cytb5**	0.35 ± 0.01	46.16 ± 0.86	0.92 ± 0.00	0.985
**Nanodiscs-cytb5**	0.29 ± 0.01	54.54 ± 0.35	1.23 ± 0.01	0.980

^a^Fixed.

### NMR experiments probing the interaction between cyt c and ^15^N-cytb_5_

**Figure 3:**
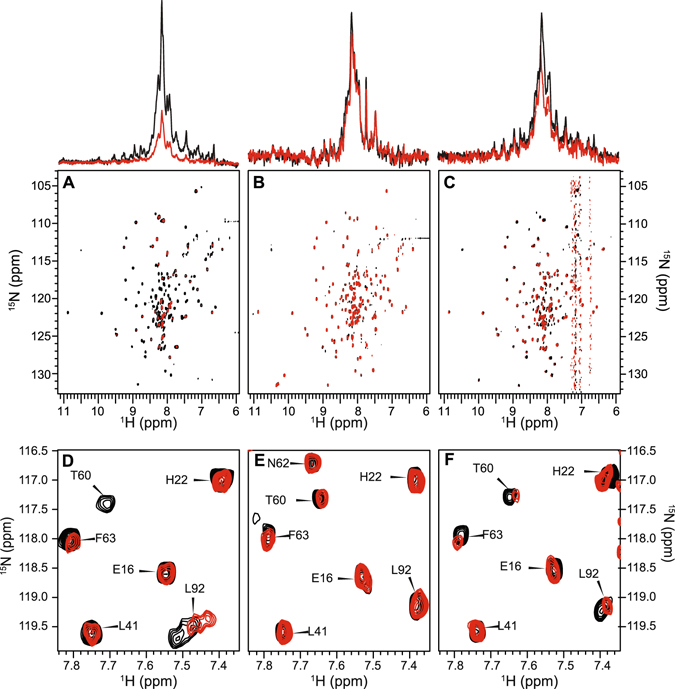
2D ^1^H-^15^N HSQC-TROSY spectra of ^15^N-labeled cyt*b*_5_ revealing the interaction between cyt*b*_5_ and cyt c. The signal intensities over the course of cyt c with the ^15^N labeled cyt*b*_5_ titration experiment are displayed in both ^15^N- edited 1D spectra (top) and accompanying 2D-TROSY-HSQC spectra (bottom) for the molar ratio of cyt*b*_5_: cyt c 1:0 (black) and 1:1 (red). (**A**) Free cyt*b*_5_ has a large loss of signal intensity throughout the course of the experiment, attributed to cytb5 aggregation in solution. (**B**) bicelle cyt*b*_5_ reconstituted in bicelles and (**C**) nanodisc cyt*b*_5_ reconstituted in nanodiscs both maintain signal intensity throughout the course of the experiment. Changes in the signal intensity and line width due to cyt*b*_5_-cyt c interactions are shown in Fig. 6 and discussed in the main text. ^1^H-^15^N HSQC spectra demonstrating chemical shift perturbations for cyt*b*_5_ in buffer (no membrane) (**D**), bicelles (**E**), or nanodiscs (**F**) in the presence of one molar equivalent of cyt c (red). As a reference, the spectrum of cyt*b*_5_ is shown without addition of cyt c (black).

Two-dimensional ^15^N/^1^H TROSY-HSQC spectra of uniformly-^15^N-labeled full-length rabbit cyt*b*_5_ were recorded to monitor changes induced by the titration of unlabeled cyt *c*. Both proteins were used in their ferric low spin oxidized forms, and titrations were carried out in a lipid-free solution, DMPC/DHPC bicelles, and DMPC-4F nanodiscs^15, 16, 18^. N-edited ^1^H 1D NMR spectra shown in Fig. 3 (top) reveal the changes in the signal intensity of cyt*b*_5_ observed from a lipid-free solution, bicelles, or nanodiscs samples as illustrated through spectra acquired over the course of the titration with unlabeled cyt *c*. In the membrane-free cyt*b*_5_ titration (Fig. 3A), there is a significant decrease in signal over the course of the first titration point (1:0, cyt*b*_5_: cyt c) to second titration point (1:0.3, cyt*b*_5_: cyt c). This decrease in signal could be attributed to a couple of things: (i) as cyt*b*_5_ aggregates, signal is lost (or reduced in intensity); (ii) similar to the trend shown in the kinetics data, membrane-free cyt*b*_5_ and cyt c are forming a static complex in solution. This larger, static complex could also lead to a decrease in tumbling speed and loss of signal. Membrane bound cyt*b*_5_ samples do not exhibit this effect and the NMR signal intensity remains relatively constant throughout the course of the titration with unlabeled cyt *c*.

The assignment of ^1^H-^15^N resonances observed in a HSQC spectrum of cyt*b*_5_ has been published previously^45^ and is shown in Figure S5. The 2D HSQC spectra provide residue specific detail about any changes in cyt*b*_5_. Upon titration with cyt *c* in this experiment, the chemical environment of these backbone amide-NH groups can be affected which can be monitored through changes observed in the 2D HSQC spectra. Two types of information can be gathered from these NMR experiments: chemical shift perturbations (Fig. 3D,E,F) and differential line broadening (Figure S6). It is expected that upon titration of cyt *c* to cyt*b*_5_, complex formation may alter the amide-NH chemical shift values of cyt*b*_5_ residues undergoing fast to intermediate time scale exchange between a free and bound forms. Chemical shift perturbations are hallmarks of transient complex formation and may be induced by direct interaction with the binding partner, as well as an overall change in protein conformation. Another possible contribution to CSPs could be due to the ring current effect from the heme groups of these two proteins. However, we expect this effect to be negligible for our system, based on the findings of Shao *et al*.^16^. Differential line broadening is indicative of a more stable, tighter complex formation due to the increase in the correlation time of the protein-protein complex. For instance, a rather flexible residue may be rigidified upon binding with cyt *c*, and its resonance will thus be broadened due to a slower tumbling rate. In addition to correlation time effects, resonance lines may be broadened due to a shift in population levels between different residue conformers^46^.

**Figure 4:**
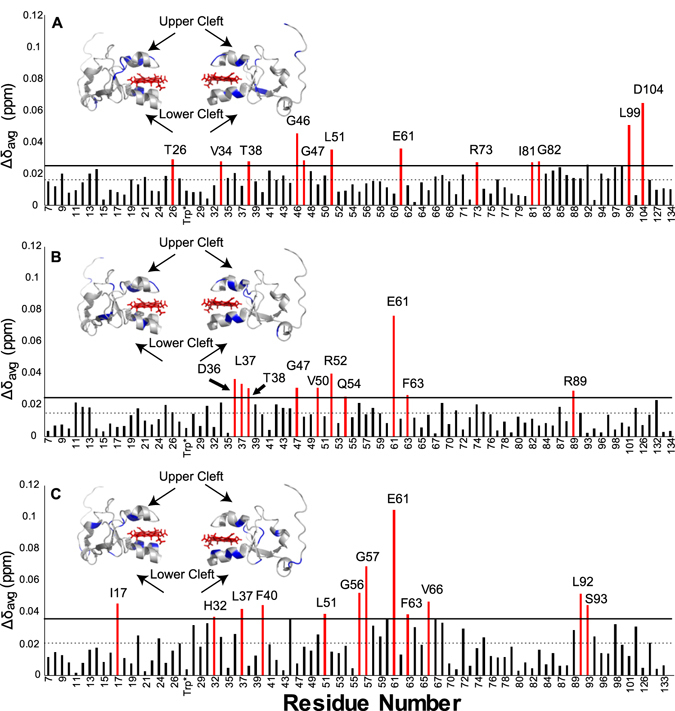
Cyt c interaction induced chemical shift perturbations of cyt*b*_5_. Reported in red are residues with CSPs greater than one standard deviation for cyt*b*_5_ in membrane-free solution (**A**), bicelles (**B**), and nanodiscs (**C**). One standard deviation above the mean is represented by the horizontal line and the mean is represented by the dashed line. The significant residues are colored in blue and are mapped onto structures of cyt*b*_5_ rotated 180° (PDB: 2M33).

After titration of cyt *c* into cyt*b*_5_, the average chemical shift perturbations (CSPs) of cyt*b*_5_ measured for lipid-free solution, bicelles, and nanodiscs are 0.016, 0.014, and 0.021 ppm, respectively. These CSPs are not very large which suggests a weak complex formation. The residues with high CSPs are widespread on the surface of the protein, which is a sign of encounter complexes present in the sample that are expected to form with electron transfer proteins^47–49^. While there are unique CSP data for each condition (lipid-free solution, bicelles, or nanodiscs), there is overlap of identified residues and a general region of cyt*b*_5_ which seems to be most affected (Fig. 4). All three conditions identify residue E61’s resonance to be highly perturbed and likely to be involved in the interactive interface of the cyt*b*_5_–cyt *c* complex. Most of the residues identified as highly perturbed are located on the upper and lower clefts surrounding the heme group of cyt*b*_5_. In the lipid-free sample, Leu99 and Asp104 were identified as highly perturbed. These residues are located in a flexible region of cyt*b*_5_ and most likely affected only in this particular sample because cyt*b*_5_ is not anchored in a lipid bilayer, unlike in bicelles or nanodiscs.

**Figure 5:**
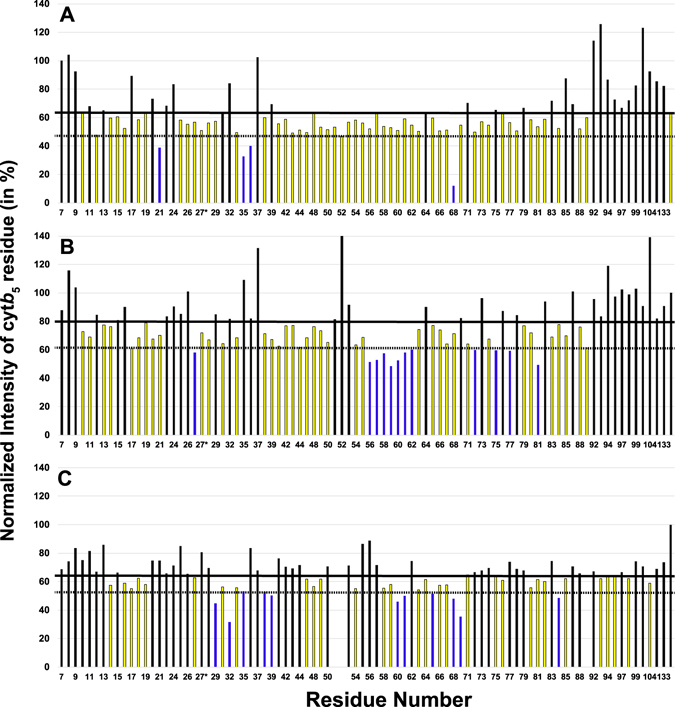
Differential line broadening reveals binding sites on cyt*b*_5_. Reported in yellow are residues with depleted signal intensity less than the mean and residues in blue have depleted signal intensity less than one standard deviation below the mean for cyt*b*_5_ in no membrane (**A**), bicelles (**B**), and nanodiscs (**C**). The thick horizontal line represents the mean and the dashed horizontal line represents on standard deviation below the mean.

**Figure 6:**
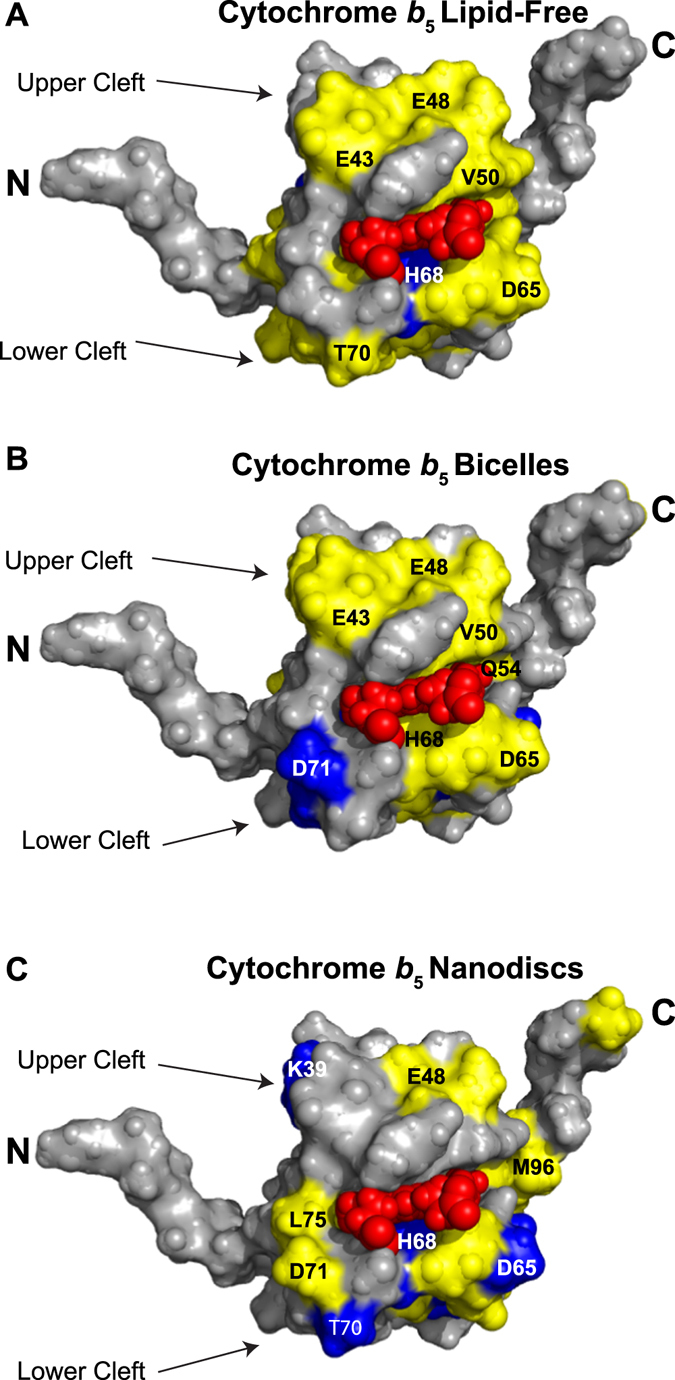
Implicated binding sites on cyt*b*_5_ mapped with differential line broadening data. The data from Fig. 5 is mapped onto a solution NMR structure (PDB: 2M33) of cyt*b*_5_ for a membrane-free solution (**A**), bicelles (**B**), and nanodiscs (**C**). Residues falling one standard deviation below the mean are colored in blue and residues falling under the mean are colored in yellow.

Differential line broadening was seen for all conditions of the cyt *c* titration into cyt*b*_5_. In this protein-protein interaction, differential line broadening is a complementary metric to CSP data to analyze the binding as it covers the timescale of the complex that falls into the time scale of ~10^-3^ s. The average relative signal intensity observed for the lipid-free solution, bicelles, and nanodiscs samples are 63.9, 79.3, and 65.1% respectively (Fig. 5). These residues are mapped onto cyt*b*_5_ structures (PDB: 2 m33) shown in Fig. 6 with lipid-free solution (Fig. 6A), bicelles (Fig. 6B), and nanodiscs (Fig. 6C). In the lipid-free solution (Fig. 5A), there is general line broadening of resonances from cyt*b*_5_ in the heme pocket with only one significant residue in the lower cleft identified, His68. The widespread line broadening is concentrated on solvent exposed residues, which can be attributed to the encounter complexes that form in redox partner pairs before the productive binding site is found.

There is consensus in affected residues from both the chemical shift perturbation data as well as the differential line broadening data. Similar areas are implicated in binding, generally the upper and lower clefts of cyt*b*_5_ which surround the heme. Differences in the two types of data can be attributed to that line broadening may be due to direct binding and stabilization of the proteins, whereas CSPs can be induced by global changes. Cyt*b*_5_ in the bicelle sample (Fig. 5B) identifies more significant residues than lipid-free cyt*b*_5_. These important residues are in the heme pocket, specifically in the lower cleft: Gly56, Gly57, Asn58, Ala59, Thr60, Glu61, Gln62. The lipid nanodisc sample (Fig. 5C) has the similar average relative peak intensity to lipid-free cyt*b*_5_ but has eleven important residues: I29, H32, T38, K39, T60, E61, D65, H68, T70, and L84. These residues are mainly clustered on the lower cleft of cyt*b*_5_; the residues L51, R52, and R89 in the nanodisc sample could not be assigned.

### NMR experiments probing the interaction between ^15^N-cyt c and cytb_5_

As with the ^15^N-labelled cyt*b*5 NMR experiments, two-dimensional ^15^N-^1^H TROSY-HSQC spectra of uniformly ^15^N-labeled equine cyt c were recorded to monitor the cyt c – cyt*b*_5_ interaction on the cyt c interface. Full-length cyt*b*_5_ in 4F-DMPC nanodiscs was used in this titration experiment based on the previous ^15^N-labeled cyt*b*_5_ NMR experiments. The ^1^H-^15^N amide resonances have been reported in the literature^50^ and the assignment is shown in Figure S2.

**Figure 7:**
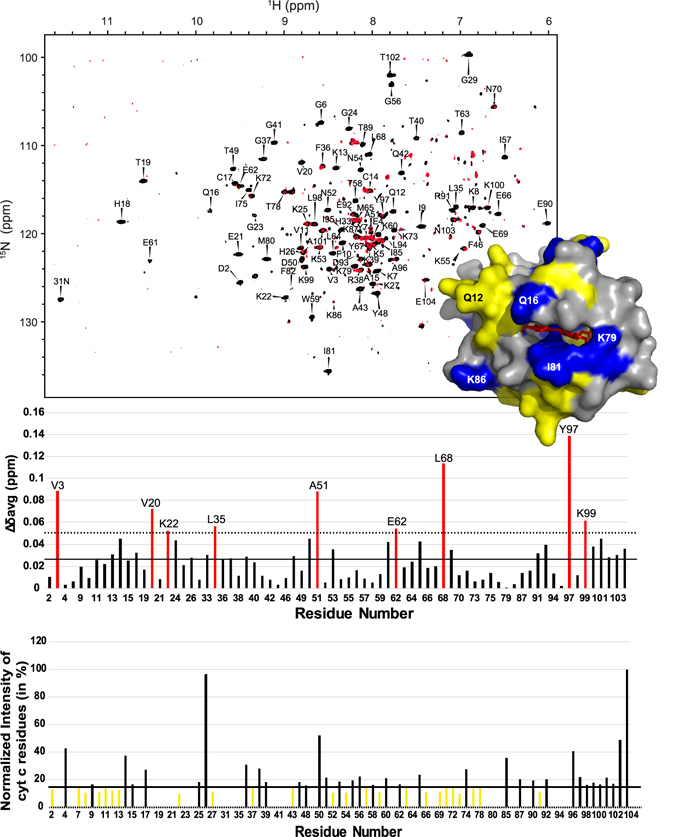
2D ^1^H-^15^N HSQC-TROSY spectra of ^15^N-labeled cyt c revealing interaction between cyt c and cyt*b*_5_. (**A**) ^1^H-^15^N spectra of ^15^N-labeled cyt c (black) and ^15^N-labeled cyt c in the presence of one molar equivalent of cyt*b*_5_ reconstituted in 4F-DMPC nanodiscs (red). The full ^15^N-labeled cyt c spectrum with resonance assignment can be found in SI Fig. 5. (**B**) Chemical shift perturbations of cyt c residues. Reported in red are residues with CSPs greater than one standard deviation. (**C**) Differential line broadening observed for cyt c residues. The thick horizontal line represents the mean, with the residues colored in yellow, while the dashed horizontal line represents one standard deviation below the mean. The inset in Fig. 8A is the crystal structure of cyt c (PDB: 1HRC) with mapping of experimentally measured differential line broadenings (data reported in 8C) for residues falling under the mean colored in yellow and residues with complete signal intensity depletion in blue.

CSPs were of similar magnitude to the chemical shifts perturbations of cyt*b*_5_, with an average value of 0.027 ppm. For the cyt c CSP calculations, the presented data (Fig. 7B) is calculated from the 1:0.6 (cyt c/cyt*b*_5_ molar ratio) rather than the 1:1 ratio as many of the residues in the 1:1 spectra have been broadened beyond detection. The nine residues which have the strongest CSPs (Val3, Val20, Lys22, Leu35, Ala51, Glu62, Leu68, Tyr97, and Lys99) are widespread throughout cyt c.

**Figure 8:**
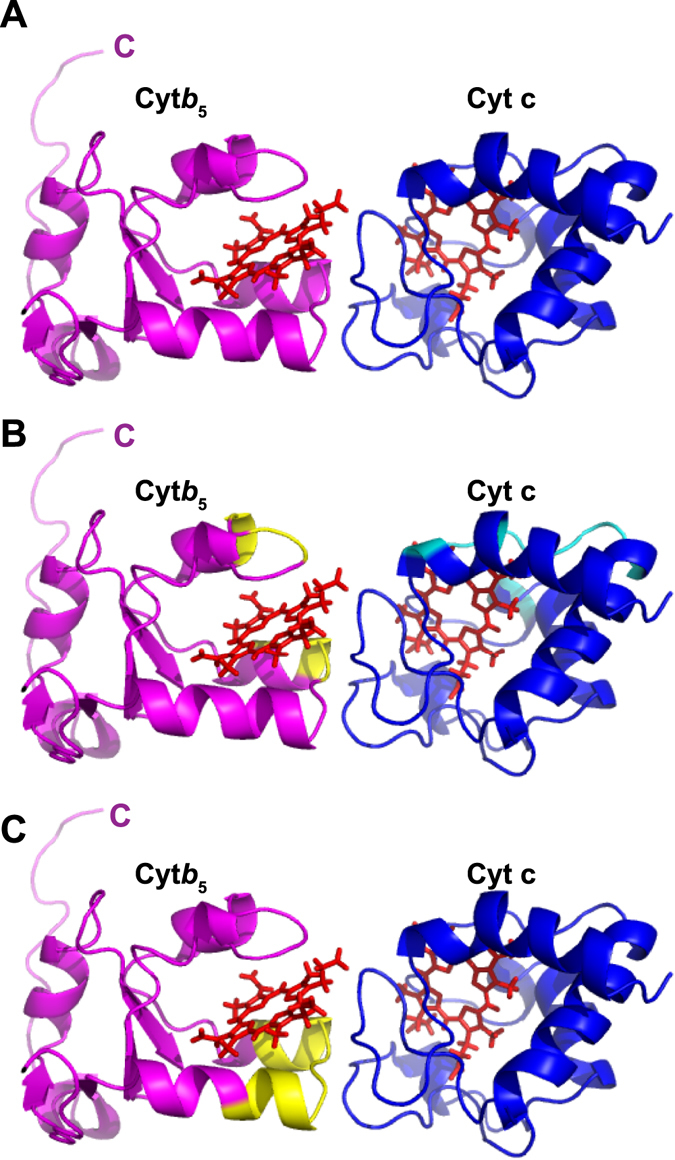
HADDOCK-generated structures reveal complex between cyt*b*_5_ and cyt c. (**A**) Using the chemical shift perturbations and differential line broadening data from the cyt*b*_5_-4F-DMPC-Nanodiscs and cyt c NMR titration experiments, a complex structure was calculated. Cyt*b*_5_ is in magenta and cyt c is in blue. (**B**) Residues implicated in the interface of cyt*b*_5_-cyt c from the literature (17) are mapped onto our HADDOCK structure; cyt*b*_5_ residues in yellow and cyt c residues in cyan. (**C**) Residues implicated in the interface of cyt*b*_5_-cytP450 from the literature (42) are mapped onto our HADDOCK structure; cyt*b*_5_ residues in yellow.

Substantial differential line broadening was seen for the titration of cyt*b*_5_ into cyt c. A complete depletion of signal was observed for 28 residues. The average intensity of the remaining residues was 17%. (Fig. 8C) These affected residues are located around in the unstructured loops surrounding the heme group of cyt c. One explanation for the dramatic decrease in peak intensity is due to a greater increase of size upon complex formation; it should be noted that cyt c alone does not bind to membrane. Viewing the complex from the 10-kDa cyt c, a 15-kDa cyt*b*_5_ molecule embedded in a ~120-kDa nanodisc causes a much bigger effect on the tumbling rate than that of cyt c on the cyt*b*_5_-ND side.

### Structural Model of the membrane-bound cytb_5_-cyt c complex

A structural complex of cyt*b*_5_-cyt c was generated using the information driven docking program HADDOCK 2.2^51, 52^ with experimental data obtained in lipid membranes (cyt c and cyt*b*_5_ in nanodiscs). NMR based chemical shift perturbation (CSPs) and differential line broadening data were used as proximity restraints to guide the docking process whereby rigid body docking follows the semi-flexible refinement and energy minimization in explicit solvent to allow the free movement of backbone and side chain atoms of the selected amino acids to improve the intermolecular packing at protein interface. Solvent-accessible residues of cyt c identified from CSPs and differential line broadening (Fig. 7) upon complex formation are selected as the active ambiguous restraints including residues Gln16, Cys17, His18, Thr19, Val20, Glu21, Gly29, Thr49, Lys79, Met80, and Ile81. Passive restraints, which are defined as solvent accessible residues around the active restraints, include Lys27, Thr28, Thr78, Phe82, and Ala83. For the cyt*b*_5_ side of the complex, active ambiguous restraints (AIRs) were defined from the cyt*b*_5_ reconstituted in nanodiscs NMR experiments (Figs 4C and 5C) including: Asp65, His68, and Thr70. The passive residues were defined as Gly67 and Ser69. Some of the residues for cyt*b*_5_ that were identified from the NMR experiments were not included in simulations as they were distant from the binding interface of proteins and were observed to penalize the HADDOCK score. Additionally, these residues are involved in significant CSPs because of the encounter complex formation formed prior to productive electron transfer complex. The HADDOCK run was performed as described under “Materials and Methods”.

Careful analysis and comparison of HADDOCK results from various clusters along with experimental data (CSPs and differential line broadening) credits cluster 1 as the most probable model for the complex (Fig. 8). From the 200 lowest energy structures, Cluster 1 is the largest (covers 75%) with better energy and Z score relative to other clusters (Table S1).

In cluster 1, the binding interface of “productive” cyt*b*_5_-cyt c complex comprises of polar and charged residues from the helical hairpin of the lower cleft of cyt*b*_5_ and the unstructured loops of cyt c. The distance between the two heme groups (Fe-Fe) was estimated as 18.5 Å and is perpendicularly oriented to each other. This is similar to the reported fluorescence quenching studies, where distance between the prosthetic groups was observed to be ~18 Å with the almost perpendicular heme planes^17^.

## Discussion

While it is highly important to determine the high-resolution dynamics-enriched structural interactions between cytP450 and its redox partners such as cyt*b*_5_, the large-size and aggregation-prone full-length cytP450 poses numerous challenges to the existing biophysical techniques. To overcome this challenge, cyt *c* has been used as a substitute for cytP450 as cyt *c* is a stable and well-behaved protein that has some important features like that of cytP450^15, 16, 18, 28^. Therefore, we used cyt *c* in this study, for the first time, to probe the complex formation between cyt*b*_5_ and cyt *c* in the presence of a lipid bilayer membrane. Both bicelles and nanodiscs were used as a model membrane in this study^43, 53^. Since bicelles are not free of detergents, and detergents are not desirable for studies on a membrane protein or a membrane-bound protein-protein complex, results obtained from bicelles were also compared with that obtained from lipid nanodiscs. A synthetic peptide (denoted as 4 F) capable of solubilizing lipids without the use of any detergents to form nanodiscs^54^ was used to form a stable lipid bilayer containing nanodisc as characterized by DLS and SEC experiments (Fig. 1). Previous studies reported the physicochemical characterization of 4 F and its use as an apolipoprotein A-I mimetic for atherosclerosis inhibition have been reported^54, 55^. NMR experiments demonstrate that the nanodiscs are suitable for structural studies by solution NMR spectroscopy. Our NMR experiments also illustrate how cyt*b*_5_ is more stable in the presence of lipids and that studies with full-length cyt*b*_5_ can be accomplished using membrane mimetics without aggregation related problems^16^.

Our study reveals that cyt*b*_5_ in a lipid-free environment, in isotropic bicelles, and nanodiscs can reduce cyt *c*. As shown in Fig. 2, stopped-flow experiments revealed that electron transfer from cyt*b*_5_ to cyt *c* occurs faster in the lipid-free cyt*b*_5_ sample over the membrane-bound cyt*b*_5_ samples. One explanation for this behavior is that the membrane-free cyt*b*_5_ can tumble faster than that in bicelles or nanodiscs, and thus able to find cyt *c* quicker to form a productive complex. This is also revealed by the higher affinity (*k*_2_ ~ 0), indicating the absence of any observable dynamic between the two proteins. The nanodiscs reconstituted cyt*b*_5_-cyt *c* sample appears to support electron transfer function better than the bicelles reconstituted cyt*b*_5_-cyt *c* sample as indicated by a quicker rate of electron transfer in the nanodiscs sample. This is possibility due to the size of the nanodisc versus the size of the bicelle – the nanodiscs are much smaller; another possible reason could be the presence of detergents in bicelles that may cause the observed slow electron transfer rate. Nanodiscs are a highly suitable system to study the interaction between the cyt*b*_5_ – cyt *c* complex as demonstrated by SEC, DLS, and NMR data. The advantageous use of nanodiscs is reflected in the functional complex formation, as well as the high stability of cyt*b*_5_, and the well-resolved NMR spectra. In comparison to the bicelle sample, the nanodiscs can both monomerize and stabilize the protein whereas the bicelles can only help to stabilize.

Many of the lipid-free cyt*b*_5_ residues implicated in the binding to cyt *c* are in two clusters: the upper and lower clefts surrounding the active site and the beta sheets at the back of cyt*b*_5_. The residues with high chemical shift perturbations in the upper and lower clefts are: Thr38 in the α2 helix, Leu51 in the α3 helix, Glu61 in the α4 helix, and Arg73 in the α5 helix. The other identified residues are: Thr26 in the loop between β2 and β3 strands, Val34 in the β3 strand, Gly46 in the β4 strand, Gly47 in the loop between β4 strand and α3 helix, Ile81 and Gly82 in the β5 strand, and Leu99 and Asp104 located in the flexible linker region between the soluble and transmembrane domains. Differential line broadening data for this sample revealed four residues: His68, one of the axial ligands for the iron in the heme; Asn21, the linker between turn 1 and β2 strand; Tyr35 and Asp36 in β3 strand. While the exact residues implicated are not all the same as reported in the previous study, the implicated residues in this sample are like previous findings^16^, with residues falling into two different clusters.

In the bicelles-reconstituted cyt*b*_5_ sample, the main cluster of residues suggested to be involved in binding to cyt *c* are found on the upper and lower clefts of the protein. Chemical shift perturbation data indicate the residues in the lower and upper clefts to be: Thr38 in the α2 helix, Val50 and Arg52 in the α3 helix, and Glu61 and Phe63 in the α4 helix. Other residues in more flexible areas of the protein are Leu37 in the linker between β3 strand and α2 helix, Glu54 in turn 3, and Arg89 in turn 4, as well as Asn36 in the β3 strand. The differential line broadening data mostly reveal residues from turn 3 to the α4 helix including Gly56, Gly57, Asp58, Ala59, Thr60, Glu61, and Asn62. The α5 helix residues Ala72, Leu75, and Lys77 were also found to be involved. Three other residues, not located in this area, that were likely to be involved in the interaction are: Ile in the α1 helix, Trp27 in the β3 sheet, and I81 in the β5 sheet. Unlike the Shao *et al*. 2003^16^ study, these residues mainly fall into one cluster surrounding the active site. The nanodisc reconstituted cyt*b*_5_ sample has more overall line broadening effects as the overall ^15^N-edited proton signal intensity decreased the most in comparison (Fig. 4). Eleven residues are indicated to be highly involved in binding: three in β3 sheet, Ile29, His32, and Tyr35; two in α2 helix, Thr38 and Lys39; three in α4 helix Thr60, Glu61, and Asp65; the axial ligand His68; Thr70 in α5 helix, and Leu84 in a loop. The chemical shift perturbation data from nanodiscs does reveal more residues, with many around the turn3 to α4 helix, similarly to the bicelles reconstituted cyt*b*_5_ data: Gly57, Asp58, Glu61, and Phe63. NMR results from both the nanodiscs and bicelles reconstituted cyt*b*_5_ also indicate residues from turn 3 to the α4 helix could play a role in the interaction between the protein in the presence of lipids as the membrane-free cyt*b*_5_ sample did not identify the residues in this region.

The binding mode of the intermolecular complex (cyt*b*_5_-cyt c) was estimated using a data driven docking approach implemented in HADDOCK. The generated structural model (Fig. 8A) presents a binding interface focused on the helical hairpin of the lower cleft of cyt*b*_5_ and the unstructured loops cyt c. The predicted electron transfer scheme generated from the cyt*b*_5_-cyt c complex structure with HARLEM^56^ shows a predicted, physiologically-feasible electron transfer path from the heme b of cyt*b*_5_ through Lys 27 to Thr 28 to heme c of cyt c. This interface was compared with that calculated in the literature by Deep *et al*.^18^. The residues implicated in binding in this study are mapped onto our complex in Fig. 8B in yellow for cyt*b*_5_ and cyan for cyt c. These results reveal a difference between our calculated structure and previously reported structures: we propose a front to front interaction as opposed to the reported side to side interaction. Comparing the results from this study on cyt*b*_5_-cyt c with that of Ahuja *et al*.^43^ on cyt*b*_5_-cytP450, we observed that the interacting interface of cyt*b*_5_ is similar and contains overlapping residues for interactions with both cytP450 and cyt c. In Fig. 8C, the residues implicated in the cyt*b*_5_-cytP450 structure by Ahuja *et al*.^43^ are mapped on our model structure, with the cyt*b*_5_ residues highlighted in yellow. The cyt*b*_5_ residues align very well with our proposed complex structure, illustrating both the importance of utilizing membrane mimetics and the viability of using cyt c as a model. The docking simulations were also performed using the active and passive residues only from the upper cleft of cyt*b*_5_. The structures generated post simulation have very low cluster size and high RMSD. Thus, it cannot be considered to be involved in intermolecular interaction. Therefore, NMR detected residues away from binding interface show that enzymes undergo multiple conformational substates.

In summary, our studies illustrate the importance and advantages of studying complex formation of membrane proteins in their native, membrane environment. Both bicelles and nanodiscs stabilize a functional cyt*b*_5_ – cyt *c* complex. To the best of our knowledge, this is the first report on the use of the 4 F peptide based nanodiscs to reconstitute a membrane protein or a protein-protein complex, while previous studies characterized the physiochemical properties and demonstrated the use of 4 F as an apoA-I-mimetic for atherosclerosis inhibition^54, 55^. With the stability of nanodiscs allowing other characterization methods through SEC and DLS measurements, along with smaller size, nanodiscs make for a better membrane mimetic. The identification of key residues mediating the cyt*b*_5_ – cyt c interaction provides important insights into the residues that drive the protein-protein complex formation. The combination of membrane systems and methods utilized in this study are also promising approaches to tackle the structural details of the more physiologically relevant cyt*b*_5_ – cytP450 and other membrane-bound electron transfer complexes.

## Experimental Procedures

### Materials and Reagents

Phosphate buffer components (potassium phosphate monobasic and dibasic) were purchased from Sigma-Aldrich (St. Louis, MO). 1,2-dihexanoyl-*sn*-glycero-3-phosphocholine (DHPC) and 1,2-dimyristoyl-*sn*-glycero-3-phosphocholine (DMPC) were purchased from Avanti Polar Lipids, Inc. (Alabaster, AL). Amino acid sequence of the 4 F peptide used to prepare lipid nanodiscs: **DWFKAFYDKV AEKFKEAF**. Equine cytochrome c was obtained through a collaborator that was prepared and purified as described previously^35^. Deuterium oxide and ^15^N Celtone Base Powder was purchased from Cambridge Isotope Laboratories (Tewksbury, MA). The 5-mm symmetrical D_2_O-matched Shigemi NMR microtubes were purchased from Shigemi, Inc (Allison Park, PA).

### Preparation of cytochrome b_5_

Full-length uniformly ^15^N-labeled and unlabeled wild-type rabbit cyt*b*_5_ was expressed and purified as described previously^10, 43, 57^. Briefly, *E. coli* C41 cells were transformed with a pLW01 plasmid containing the cytb5 gene. The cells were grown in LB medium to an OD of 1 at 600 nm. The culture was diluted 100-fold into 10 mL of ^15^N-Celtone medium. This culture was grown at 35 °C with shaking at 250 rpm until an OD of 1 at 600 nm was achieved. The cells were pelleted and resuspended in 10 mL of fresh ^15^N-Celtone medium. The resuspended cell culture was added to the final 1 L of culture minimum medium. Isopropyl β-D-thiogalactopyranoside was added to a final concentration of 10 μM, and incubation was continued for 20 h, at which time the cells were harvested. ^15^N cytochrome *b*_5_ was purified as described previously^58^.

### Preparation of cytochrome c

Full-length uniformly ^15^N-labeled cyt *c* was expressed and purified using a procedure as reported in the literature (34).

### Preparation of bicelles

A DMPC/DHPC isotropic bicelle (*q* = [DMPC]/[DHPC] = 0.25) was prepared by mixing the appropriate amount of DMPC and DHPC in chloroform. The mixture was vortexed and dried under nitrogen to make a thin film, which was further dried under vacuum overnight at room temperature. The film was then hydrated in 10 mM potassium phosphate buffer, pH 7.4 to a concentration of 5% w/v.

### Preparation of nanodiscs

DMPC powder was suspended into buffer A (10 mM potassium phosphate, pH 7.4) to make a stock solution at 20 mg/mL. The 4 F peptide was dissolved in buffer A to make a stock solution at 10 mg/mL. The DMPC stock solution was vortexed and sonicated three times for 30 s each to create a suspension, and vortexed thoroughly immediately before use. The stock solution was mixed together at a peptide:lipid ratio of 1:1.5% w/w and incubated at 37 °C o/n with slow agitation. The nanodiscs were purified by size exclusion chromatography (SEC). A Superdex 200 Increase 300/10 GL column was operated on an AKTA purifier (GE Healthcare, Freiburg, Germany).

### NMR experiments

NMR experiments were performed at 298 K on a Bruker Avance II 600 MHz spectrometer equipped with a cryoprobe. 2D ^15^N/^1^H TROSY HSQC spectra were recorded from 0.1 mM ^15^N-cyt*b*_5_ in buffer A, bicelles containing ^15^N-cyt*b*_5_, or nanodiscs containing ^15^N-cyt*b*_5_. All buffer and nanodisc samples were in buffer A and contained ^15^N-cyt*b*_5_ at a concentration around 0.1 mM. Cyt*b*_5_ was incubated with a 5% v/v solution of DMPC/DHPC. Cyt*b*_5_ was incubated with 4F-DMPC-Nanodiscs at a ratio of 1:1.

All NMR spectra were recorded with 64 scans and 256 t1 increments. Data was processed using TopSpin 2.0 (Bruker) and analyzed with Sparky (Goddard). The previously reported cyt*b*_5_ backbone chemical shift assignments were used in this study^45^. The weighted amide chemical shift perturbation (Δδ_avg_) was calculated using the following equation:

1 $${\rm{\Delta }}{\delta }_{avg}=\sqrt{{({\rm{\Delta }}\delta N\times \frac{{F}_{2}SW}{{F}_{1}SW})}^{2}+{\rm{\Delta }}\delta {H}^{2}}$$

where Δδ N and Δδ H are the changes in the chemical shifts of amide nitrogen-15 and amide-proton respectively, while F_1_SW and F_2_SW represent the spectral width in the first and second dimensions respectively; chemical shift values are given in ppm^59, 60^.

### Stopped Flow Kinetics

All experiments were performed at 25 °C under anaerobic conditions using a Hi-Tech SF61DX2 stopped-flow spectrophotometer (Bradford-on-Avon, UK) housed in an anaerobic Belle Technology glove box (Weymouth, UK). The buffer was purged with nitrogen gas for 30 minutes for deoxygenation prior to being transferred to the glove box. All protein solutions were incubated overnight at 4 °C in the glove box to eliminate oxygen. For measuring the kinetics of cyt c reduction by cyt*b*_5_, cyt*b*_5_ was pre-reduced anaerobically as described below and then loaded in syringe 3 of the stopped flow, and syringe 2 was loaded with ferric cyt c. Spectra were recorded in the photodiode array mode between the wavelength of 300 and 750 nm after mixing equal volumes of the 12 μM cyt*b*_5_ and the 13.2 μM cyt *c* containing solutions resulting in final concentrations of 6 μM cyt*b*_5_ and 6.6 μM cyt c. Each set of samples (free non-membrane bound cyt*b*_5_, bicelle cyt*b*_5_, nanodisc cyt*b*_5_) was measured over different amounts of time between 0.8 and 20 s.

### Data analysis and kinetic modeling

Spectra were collected and Singular Value Decomposition (SVD) was applied in the differential spectrum using an algorithm written in Matlab (The Mathworks Inc., v.R2016b)^61^. Before SVD, Savitzky-Golay smoothing and peak-to-area normalization were performed on all the recorded spectra to increase signal-to-noise ratio. SVD analysis was then performed using the built-in Matlab function, obtaining the corresponding kinetic and spectral eigenvectors. The number of principal components was chosen by visualization of the scree plot (Figure SI6, Supplemental Information), as well as considering the variance covered by the selected eigenvectors. The fraction of variance was computed as following:

2 $${f}_{q}=\frac{{\sigma }_{q}^{2}}{{\sum }_{t=1}^{m}{\sigma }_{t}^{2}},\quad q=1,\ldots ,m$$

where *f*_*q*_ represents the fraction of the expression level contributed by the *q* ^*th*^ eigenvector, ${\sigma }_{q}^{2}$ is the variance of the *q* ^*th*^ eigenvector, and ${\sigma }_{t}^{2}$ the total variance. In all the performed experiments, two eigenvectors were selected (PC1 and PC2), covering 70–90% of the total variance.

### Kinetic Modeling and Simulations

For the three systems (lipid-free, bicelles and nanodiscs), based on the evolution over time and peak maxima, PC1 was assigned to cyt *c*, and PC2 to cyt*b*_5_. The final concentration of ferrous cytochrome *c* was computed by measuring the absorbance at ~550 nm and using ε = 21.2 mM^−1^ cm^−1^. Cyt*b*_5_ was considered to be completely oxidized at the end of the experiment. The kinetics model that describe the oxidoreductive reaction is depicted in Fig. 2. The differential equations to model the electron transfer kinetics were parameterized using the NonlinearModelFit function with 1/Y weighting in Mathematica 11.0 (Wolfram Research, Champagne, IL). When fitting parameters, the ParametricNDSolveValue function was used for numerical solutions of the differential equations with MaxSteps → 100,000 and PrecisionGoal → ∞.

### Titration of cytb_5_ by Dithionite under Anaerobic Conditions

Cyt*b*_5_ (12 μM) was titrated with a standardized sodium dithionite solution under anaerobic conditions while monitoring the UV-visible spectra with a Cary 4000 spectrometer between 300 and 700 nm. The stock solution of cyt*b*_5_ was incubated overnight at 4 °C in an anaerobic Belle Technology glove box (Hi-Tech, Salisbury, UK) to remove oxygen. The titrant (sodium dithionite solution) was prepared in the glove box in oxygen free buffer, and its concentration was calculated using an extinction coefficient of 8.04 mm^−1^ cm^−1^ at 315 nm. The titration was carried out in a tonometer, a homemade anaerobic titration apparatus.

### Calculation of a membrane-bound cytb_5_-cyt c complex using NMR data

The HADDOCK (High Ambiguity Driven protein-protein DOCKing) docking software^51, 52^ was used to calculate the structures of the cyt*b*_5_-cyt c complex based on experimental NMR restraints. The HADDOCK algorithm includes three consecutive steps: (1) rigid body docking in which the two molecules are rotated and translated randomly in turn to minimize intermolecular energy; (2) simulated annealing of structures in which annealing in torsion angle space is performed to refine the orientation of the proteins and the side chains and/or backbones of the interface residues; and (3) solvent refinement in which the structures are further refined in an 8.0 Ǻ shell of TIP3P water molecules. The starting structures for this docking were the solution NMR structure of rabbit cyt*b*_5_ (PDB: 2M33) and the crystal structure of horse cyt c (PDB: 1 HRC). Ligand topology and parameter files were generated using PRODRG2 server^62^. Defined ambiguous restraints were generated from NMR experiments performed in this paper, both active and passive residues with solvent accessibility from both proteins. 1000 structures were generated in the rigid body docking step, followed by simulated annealing of the 200 lowest energy structures from the last step, and 200 structures were selected for solvent refinement. The resulting 200 final structures were analyzed and grouped into clusters based on the backbone root mean square deviation values. Molecular structures of the complexes were viewed and graphed using PyMOL.

### Data availability

Most of the data generated or analyzed during this study are included in this article (and in the Supporting Information files) and other datasets are available from the corresponding author on reasonable request.

## Electronic supplementary material

Supplementary Information
