# Supplementary material for: Kinetic and Structural Characterization of the Effects of Membrane on the Complex of Cytochrome b5 and Cytochrome c
Source: Sci Rep. 2017 Aug 10;7:7793. doi: 10.1038/s41598-017-08130-7 (PMC5552742; doi:10.1038/s41598-017-08130-7)
Supplement: Supplementary file 1 — Supplementary Information [file 41598_2017_8130_MOESM1_ESM.docx]

**Kinetic and Structural Characterization of the Effects of Membrane on the Complex of Cytochrome *b*_5_ and Cytochrome *c* **

Katherine A. Gentry^1^, Elke Prade^1,2^, Carlo Barnaba^1,2^, Meng Zhang^2^, Mukesh Mahajan^1,2^, Sang-Choul Im^3^, G. M. Anantharamaiah,^4^ Satoshi Nagao,^5^ Lucy Waskell^3^, Ayyalusamy Ramamoorthy^1,2^

^1^ Biophysics Program and ^2^Department of Chemistry, University of Michigan, Ann Arbor, MI, 48109, USA and ^3^Department of Anesthesiology, University of Michigan, and Veterans Affairs Medical Center, Ann Arbor, Michigan 48105, ^4^Department of Medicine, UAB Medical Center, Birmingham, Alabama 35294. ^5^Graduate School of Material Science, Nara Institute of Science and Technology, 8916-5 Takayama, Ikoma, Nara 630-0192, Japan

SP|P00004|CYC_HORSE ------------------------------------------------------------

SP|P00178|CP2B4_RABIT MEFSLLLLLAFLAGLLLLLFRGHPKAHGRLPPGPSPLPVLGNLLQMDRKGLLRSFLRLRE

Consensus/80% ............................................................

SP|P00004|CYC_HORSE ------------------------------------------------------------

SP|P00178|CP2B4_RABIT KYGDVFTVYLGSRPVVVLCGTDAIREALVDQAEAFSGRGKIAVVDPIFQGYGVIFANGER

Consensus/80% ............................................................

SP|P00004|CYC_HORSE -----------**M**G**DV**E**KGKK**I---**FVQKCAQCHT**V**EK**GG**K**----**HKTG**PN**LH**--------

SP|P00178|CP2B4_RABIT WRALRRFSLAT**M**R**DF**G**MGKR**SVEE**RIQEEARCLV**E**EL**RK**S**KGAL**LDNT**LL**FH**SITSNIIC

Consensus/80% ...........b.-h.bt++....blppptpsbs.-b..p....bpss..b+........

SP|P00004|CYC_HORSE -**GLFGRKT**----------------------------------------------------

SP|P00178|CP2B4_RABIT S**IVFGKRF**DYKDPVFLRLLDLFFQSFSLISSFSSQVFELFPGFLKHFPGTHRQIYRNLQE

Consensus/80% .hlat++h....................................................

SP|P00004|CYC_HORSE ------------------------------------------------------------

SP|P00178|CP2B4_RABIT INTFIGQSVEKHRATLDPSNPRDFIDVYLLRMEKDKSDPSSEFHHQNLILTVLSLFFAGT

Consensus/80% ............................................................

SP|P00004|CYC_HORSE ------------------------------------------------------------

SP|P00178|CP2B4_RABIT ETTSTTLRYGFLLMLKYPHVTERVQKEIEQVIGSHRPPALDDRAKMPYTDAVIHEIQRLG

Consensus/80% ............................................................

SP|P00004|CYC_HORSE --------------**GQA**P**GFTYTDANKNKGIT**W**K**E**ET**LM**EYLENPKKY**I**PGTKM**I**FAGIK**

SP|P00178|CP2B4_RABIT DLIPFGVPHTVTKD**TQF**R**GYVIPKNTEVFPVL**S**S**A**LH**DP**RYFETPNTF**N**PGHFL**D**ANGAL**

Consensus/80% ..............sph.tasbspsspsbslh.p.bp..pab-ssppa.stpbb.hsthb

SP|P00004|CYC_HORSE **KKTE**------------------**REDLIAYLKKATNE**------------------------

SP|P00178|CP2B4_RABIT **KRNE**GFMPFSLGKRICLGEGIA**RTELFLFFTTILQN**FSIASPVPPEDIDLTPRESGVGNV

Consensus/80% ++s-..................+p-lbhabpphhpp........................

SP|P00004|CYC_HORSE -----------

SP|P00178|CP2B4_RABIT PPSYQIRFLAR

Consensus/80% ...........

**Figure S1.** Sequence alignment of cytochrome c and cytochrome P450 reveals amino acid sequence similarity. Horse cytochrome *c* (top) and rabbit cytochrome P450 2B4 are aligned using CHROMA. Positive residues are highlighted in purple, negative residues are highlighted in cyan, serine and threonine are given in cyan text, aliphatic residues are highlighted in yellow, aromatic residues are highlighted in orange.


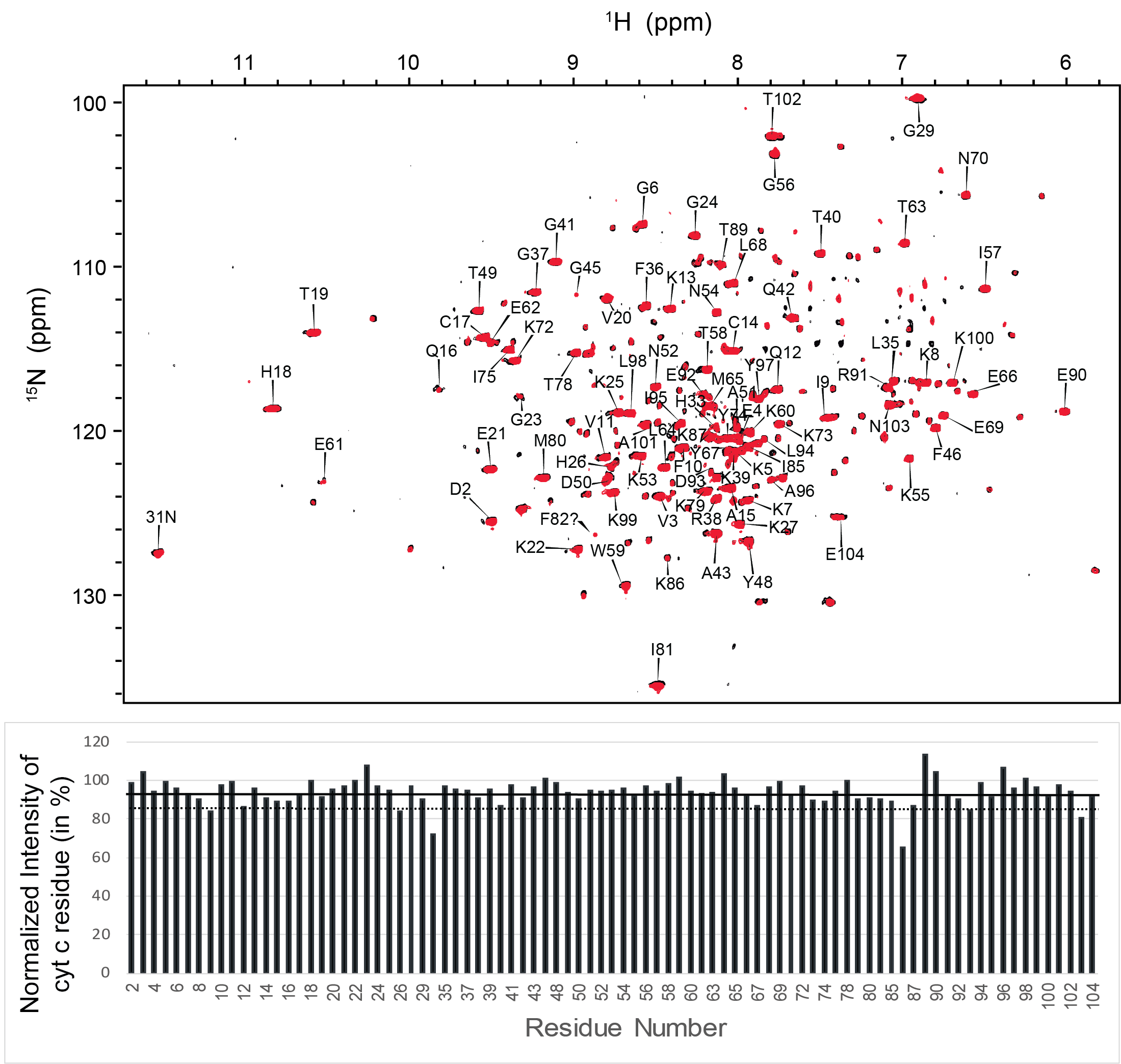


**Figure S2.** Cytochrome c does not interact with the 4F-DMPC nanodiscs. The 2D ^1^H-^15^N TROSY HSQC spectra is shown with a reference spectra of ^15^N-cyt c (in black) and with ^15^N-cyt c in the presence of 1 molar equivalent of 4F-DMPC nanodiscs. Differential line broadening for the cyt c are shown below with the bold horizontal representing the mean and the dashed line representing one standard deviation below the mean. There are no chemical shift perturbations of cyt c in this experiment.


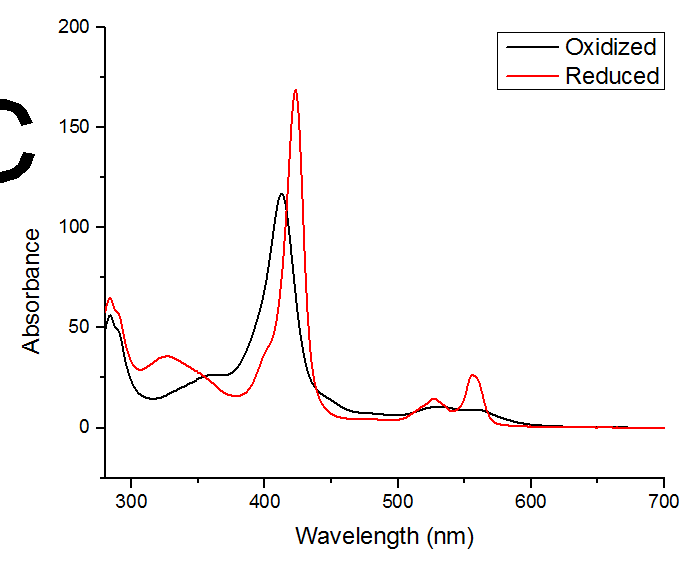


**Figure S3.** UV absorption profiles of cyt*b*_5_ in lipid-free solution from oxidized to reduced. Oxidized cyt*b*_5_ was titrated with sodium dithionate to reduced cyt*b*_5_ (red).


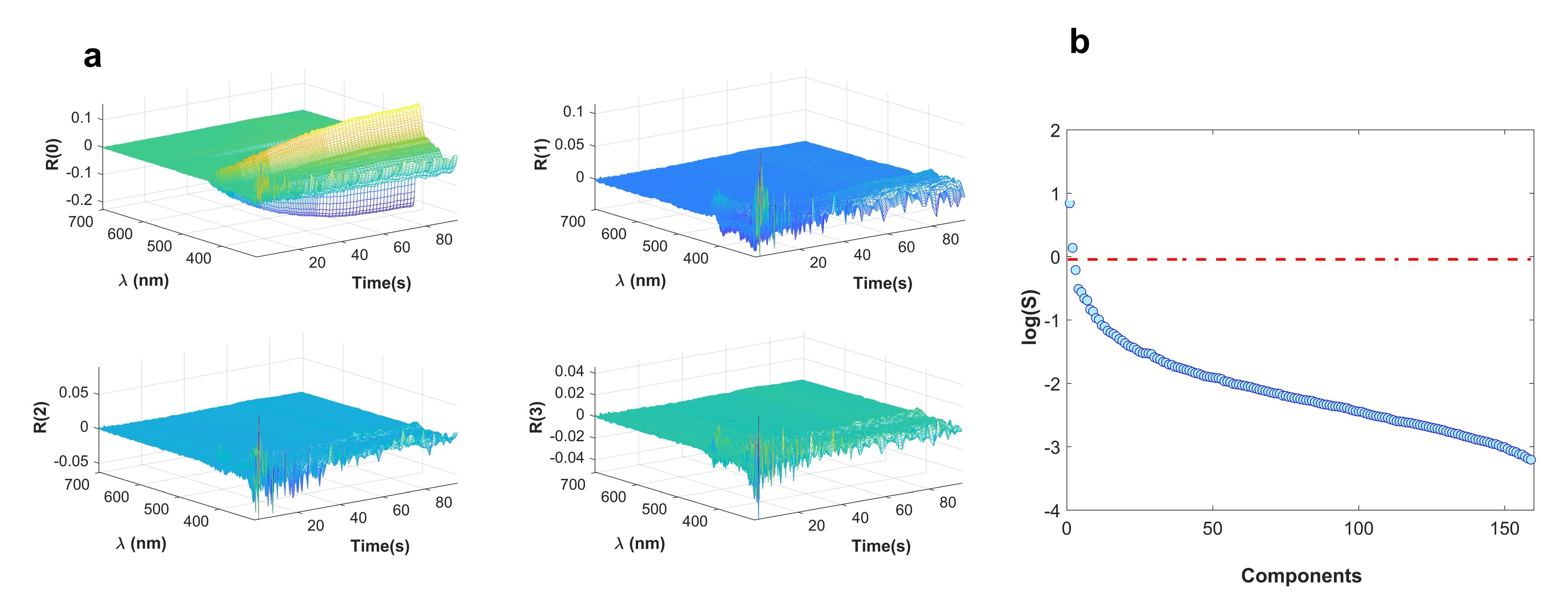


**Figure S4.** Results from SVD analysis applied to time-dependent spectra of electron transfer reaction in cyt*b*_5_-cyt c complex reconstituted in bicelles. a) raw difference spectrum (R0), and subtraction of first (R1), second (R2) and third (R3) components; b) scree plot of singular values (S), showing the components considered above background (as indicated by the red dashed line). As from both graphs, we assumed that any residual signal after subtraction of PC1 (that is R1) and PC2 (that is R2) cannot be spectrally resolved, and thus R2, R3, and the remaining components (R_i_; i>3) were considered as indistinguishable from noise.


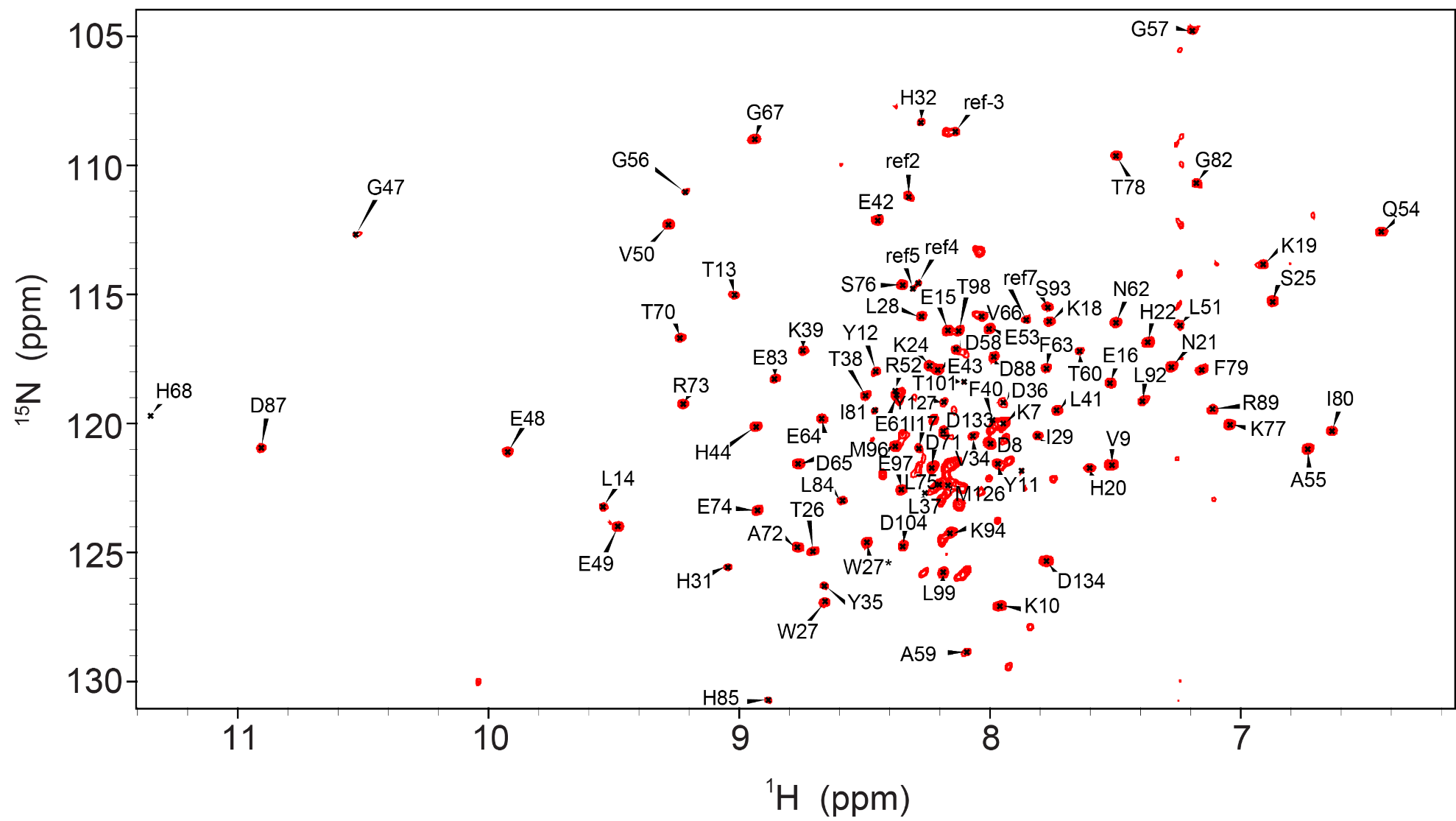


**Figure S5.** 2D TROSY-HSQC NMR spectrum is of full-length cyt*b*_5_ reconstituted in 4F-DMPC Nanodiscs. Assignment of ^1^H-^15^N resonances are included.


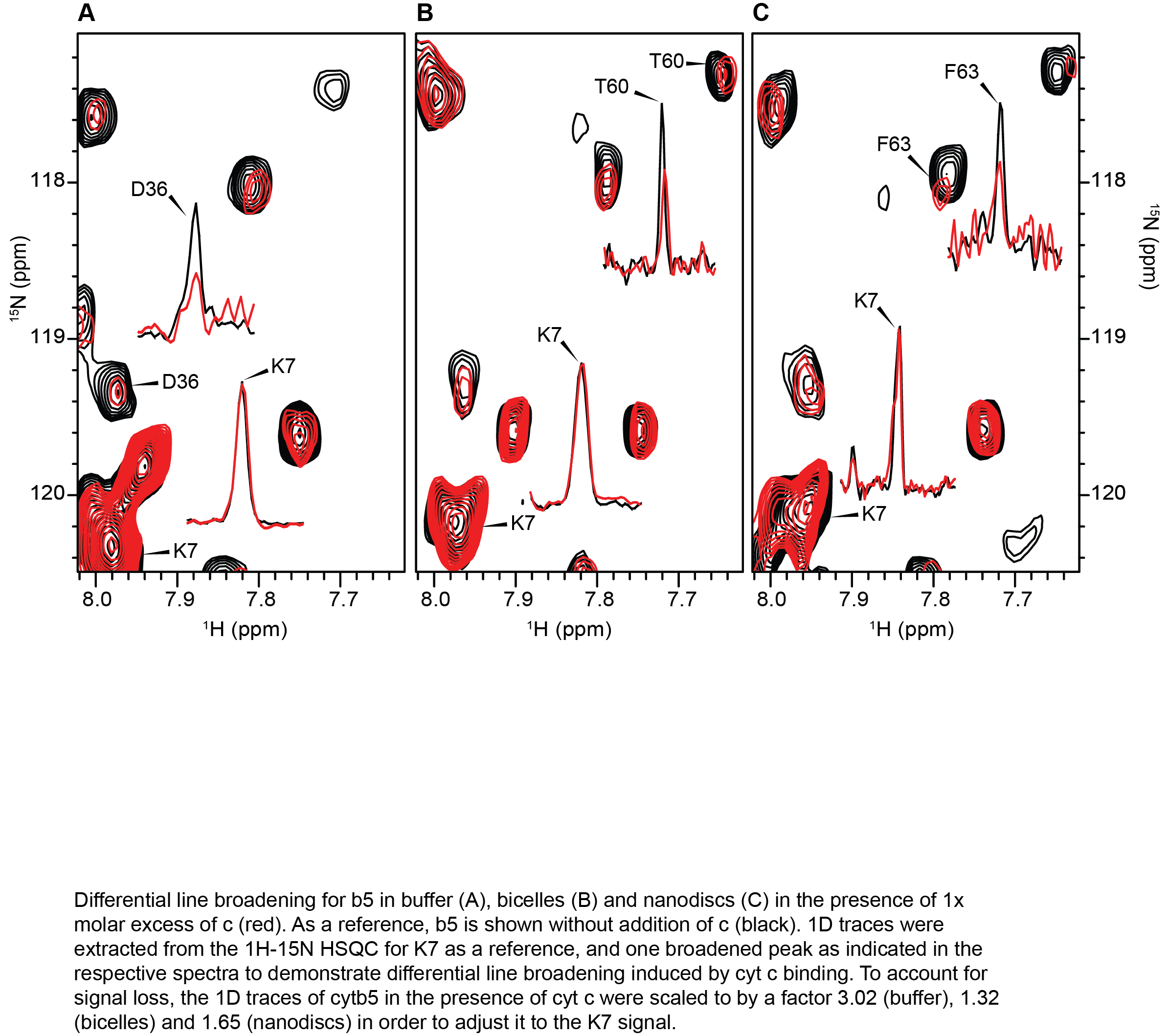


**Figure S6.** Differential line broadening for cyt*b*_5_ in buffer (no membrane) (A), bicelles (B), or nanodiscs (C) in the presence of 1 molar equivalent of cyt c (red). As a reference, cyt*b*_5_ is shown without the addition of cyt c (black). 1D spectral lines were extracted from the 2D ^1^H-^15^N TROSY HSQC spectra for residue K7 as a reference, and one of the broadened peaks as indicated in the respective spectra to demonstrate differential line broadening induced by cyt c binding with cyt*b*_5_. To account for the loss of signal intensity, the 1D traces of cyt*b*_5_ in the presence of cyt c were scaled-up a factor of 3.02 (buffer), 1.32 (bicelles), and 1.65 (nanodiscs) in order to compare with the peak from K7.

**Table S1.** Energy statistics for lowest energy cluster of the complex between cyt*b*_5_ and cyt c generated from HADDOCK.

| **Parameters** | Cluster 1 |
| --- | --- |
| Backbone r.m.s.d. (Å) Van der Waals energy Electrostatic energy Desolvation energy Restraints violation energy Buried surface area | 2.1 +/- 1.3 -22.5 +/- 2.8  -282.2 +/- 16.0  11.8 +/- 4.3 1.4 +/- 1.24 762.3 +/- 71.5 |
